# Lower Prevalence Countries Outside of South-Eastern Africa Now Have the Fastest Growing HIV Epidemic

**DOI:** 10.1093/ofid/ofae318

**Published:** 2024-06-06

**Authors:** Toby Pepperrell, Andrew Hill, Samuel Cross

**Affiliations:** School of Medicine and Veterinary Medicine, University of Edinburgh, Edinburgh, UK; NHS Greater Glasgow and Clyde, NHS Scotland, Glasgow, UK; Department of Translational Medicine, Liverpool University, Liverpool, UK

**Keywords:** ART, epidemic, HIV, incidence, prevalence

## Abstract

The United Nations Program on HIV/AIDS (UNAIDS) targets aim to reduce new HIV infections below 370 000 annually by 2025. However, there were 1.3 million new HIV infections worldwide in 2022. We collected and analyzed data for key variables of the HIV epidemic from UNAIDS and supplemented by PUBMED/EMBASE searches and national reports. A total of 53% of the HIV infections worldwide were in 14 high-prevalence countries in Southern/East Africa—where most of the funding for treatment and prevention is allocated—versus 47% in 54 low-prevalence countries. In 2022, there were more new HIV infections (770 000 vs 468 000), more HIV-related deaths (383 000 vs 225 000), higher rates of mother-to-child transmissions (16% vs 9%) and lower antiretroviral therapy coverage (67% vs 83%) in low-prevalence countries versus high-prevalence countries. To achieve UNAIDS annual new infections target for 2025, ART coverage needs to be optimized worldwide, and preexposure prophylaxis coverage expanded to 74 million people, versus 2.5 million currently treated.

## Teaser

Most of the funding for treatment and prevention of HIV is allocated to countries in Southern/Eastern Africa with the highest prevalence, but low incidence. Expansion of resources to countries with low prevalence is vital to achieving United Nations Program on HIV/AIDS targets for 2025.

## BACKGROUND

The United Nations Program on HIV/AIDS (UNAIDS) 95-95-95 targets for HIV delineate the objective of diagnosing 95% of people with HIV (PWH), ensuring that 95% of people diagnosed receive antiretroviral therapy (ART), and achieving viral suppression in 95% of people treated by the year 2025. In June 2021, UNAIDS key prevention goals were updated, aiming for <370 000 new infections worldwide by 2025. To reach this goal, UNAIDS advocates for the implementation of treatment as prevention and preexposure prophylaxis (PrEP), targeting adolescents and risk groups.

Treatment as prevention has proven effective in reducing incidence, demonstrated by notable progress across South-Eastern African nations targeted for their high prevalence. Through an intensive internationally-funded response and ART coverage >90%, Eswatini, Botswana, Lesotho, Zimbabwe, and Malawi have some of the lowest infection rates in the world, all below 2% [1]. The availability of generic antiretrovirals has made this possible. Current first-line ART, combination tenofovir-lamivudine-dolutegravir (TLD), can now be purchased generically for approximately 40–60 USD per person per year (PPPY), but only for countries included in voluntary licensing agreements. The voluntary license for dolutegravir has unclear rationale for national access, including all low- and middle-income countries by arbitrary cutoff of gross domestic product (GDP), rather than those deemed unable to afford patented medications on top of delivery expenses or appropriate socioeconomic analysis [2]. Despite the existence of affordable and effective drugs, there were 1.3 million new HIV infections worldwide in 2022, and the rate of incidence reduction has been far below that needed to meet 2025 targets [1]. To improve progress toward 95-95-95, decisions around ART licensing and health system funding need to be equitably needs driven, rather than precedent based.

Successful treatment programmes are only possible with strong funding for sexual health services to be free at the point of care so that vulnerable people can be reached and retained by clinics. Otherwise, people will often only return to clinic when they become unwell, meaning they remain virologically unsuppressed and therefore possibly infectious. Socioeconomic factors play a huge role in the likelihood of virologic suppression: poverty (often involving an inability to get to the clinic), food insecurity, drug and alcohol problems, poor mental health, and childcare issues. PWH rely on interpersonal support such as counselling to navigate treatment. The extra costs of meaningful social support programmes to ensure high suppression rates emphasize the importance of nonexclusive antiretroviral licensing, especially for middle-income countries where drugs have little impact on incidence until they can be accessed through the Indian generics market [3]. This has resulted in Colombia challenging ViiV's exclusive licensing of dolutegravir [4].

Although efforts toward the 95-95-95 goals have potential to be successful in many high-income or high-prevalence nations with large, targeted test-and-treat or PrEP campaigns, many nations will not achieve these goals on the current trajectory. The global HIV epidemic may endure at its current size because of limited capacity for effective programs in countries excluded from access agreements or ineligible for significant external support. It is important to understand the global distribution of new HIV infections, and how to mobilize truly efficacious prevention campaigns. This paper will show which areas have the greatest contribution to global incidence and mortality of HIV to explore the implications in terms of how access efforts and international funding can be more equitably directed. To contextualize the manageable scale of the efforts needed to move toward 95-95-95 if the correct priorities are set, we will also calculate global revenues of the main HIV pharmaceutical companies.

## METHODS

The UNAIDSinfo database provides estimates for a wide variety of epidemiological data, including HIV prevalence, epidemic size, ART coverage, PrEP coverage, numbers of new HIV infections, and HIV/AIDS-related deaths, as well as mother-to-child transmission (MTCT) [1]. We gathered UNAIDS data from 2023. Where UNAIDS data were not available, data were collected from national databases and reports found in specific PubMed searches, referenced in Appendix 1. Certain metrics like PrEP coverage were more poorly reported than metrics such as incidence or prevalence—where relevant, data scarcity was reported alongside results.

Countries included in this study had an epidemic size of more than 40 000 PWH, as reporting was worse for nations with smaller epidemics and prevention campaigns were not anticipated to be fully augmented. They were categorized as higher (≥3.5%) or lower (<3.5%) prevalence countries (HPCs and LPCs). This threshold split the global population of PWH roughly in half to assess weighting of disease and response between the 2 groups, with the higher prevalence group accounting for 53% and the lower prevalence group accounting for 47%.

Data were analyzed for ART coverage, PrEP uptake, new infections, mortality rate, and MTCT by generating summary statistics for HIV metrics. Mortality rate was calculated from deaths divided by epidemic size. Epidemic growth rate was calculated by new infections divided by epidemic size. This was chosen over number of new infections because it provides a ratio of transmissibility. Bubble plots were generated on Excel and were weighted by epidemic size to describe the relationship between HPCs and LPCs for each HIV metric. Quadratic trends were generated using Excel to demonstrate the relationship between HPCs and LPCs on the bubble graphs. Maps were generated through uploading the database to DataWrapper.

Number needed to treat with oral PrEP to prevent 1 infection was found to be 60 people in previous work [2]. This figure was used assess the necessary scale of PrEP coverage to reach incidence goals.

Annual revenue data for GSK, Gilead Pharmaceuticals, and Johnson & Johnson were collated from the quarterly financial reports [5–7].

## RESULTS

### Prevalence

**Figure 1.**
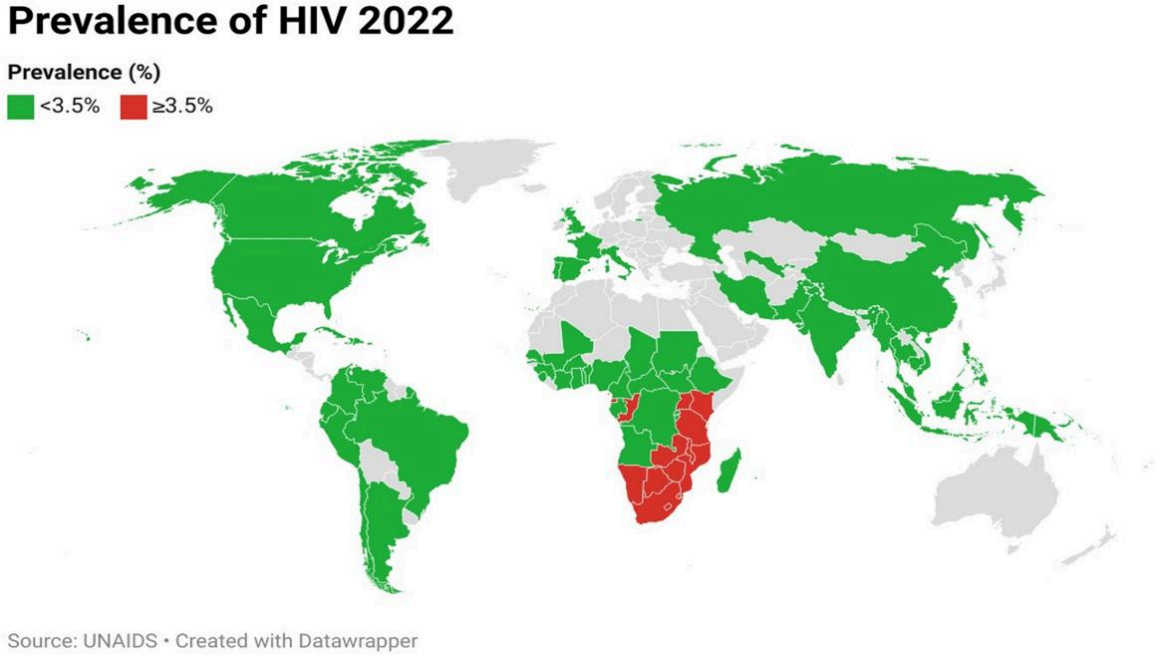
Prevalence of HIV by country. High-prevalence countries (HPCs) (≥3.5%) vs low prevalence countries (LPCs) (<3.5%).

Overall, there were 37 million PWH in the 68 countries included in this study. There were 19.5 million PWH across 14 HPCs, comprising 53% of the global population. All 14 HPCs were in South-Eastern Africa (Figure 1). Fifty-four LPCs were spread across the remainder of the globe (Figure 1), comprising 17.5 million infections, 47% of total HIV infections worldwide. Table 1 illustrates the key differences between the HPCs and LPCs in this study.

**Table 1.** Key Epidemiological Statistics for HPCs vs LPCs in 2023

	Higher Prevalence >3.5% (n = 14)	Lower Prevalence <3.5% (n = 54)	Ratio Higher:lower
Epidemic size (n)	19 462 000	17 459 263	
ART coverage	81.6%	67.4%	1.21
Mortality	225 100	383 190	0.59
New infections	468 400	770 703	0.61
Annual epidemic growth	2.41%	4.41%	0.55
PrEP initiations	1 323 493 (n = 12)	1 174 539 (n = 42)	1.13
MTCT	9%	16% (n = 43)	0.56

Abbreviations: ART, antiretroviral therapy; MTCT, mother-to-child-transmission; PrEP, preexposure prophylaxis.

### Epidemic Growth

Overall, there were 1 238 000 new infections across the countries included in this study in 2022 (Table 1). Despite a smaller total epidemic size, there were more new HIV infections in LPCs than in HPCs (770 000 vs 468 000) (Table 1), whereas the number of new infections in upper-middle or high-income countries was 375 917. The rate of HIV epidemic growth (new infections/epidemic size) was 4.4% in LPCs versus 2.6% in HPCs, showing greater relative rise in HIV infections across LPCs as well as a higher total number of new cases (Table 1).

**Figure 2.**
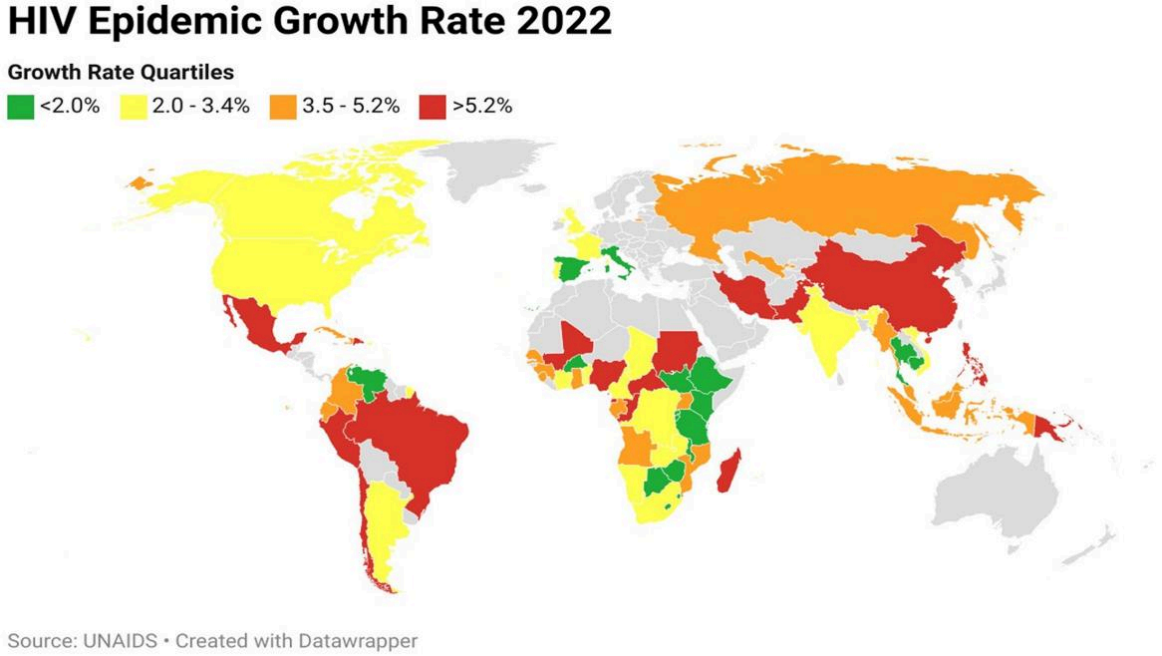
Epidemic growth rate by country by quartile. Epidemic growth rate is the number of new infections divided by epidemic size in 2022.

Figure 2 shows epidemic growth rate across the globe. Most of the nations with an epidemic growth rate >5.2% were in Central and South America, Central Africa, and Central and East Asia. The majority of HPCs were in the lower 2 quartiles (10 countries), suggesting successful implementation of prevention strategies. The HPCs in higher growth rate quartiles, such as Congo and Mozambique, are outliers because of political circumstances and so do not break this association.

**Figure 3.**
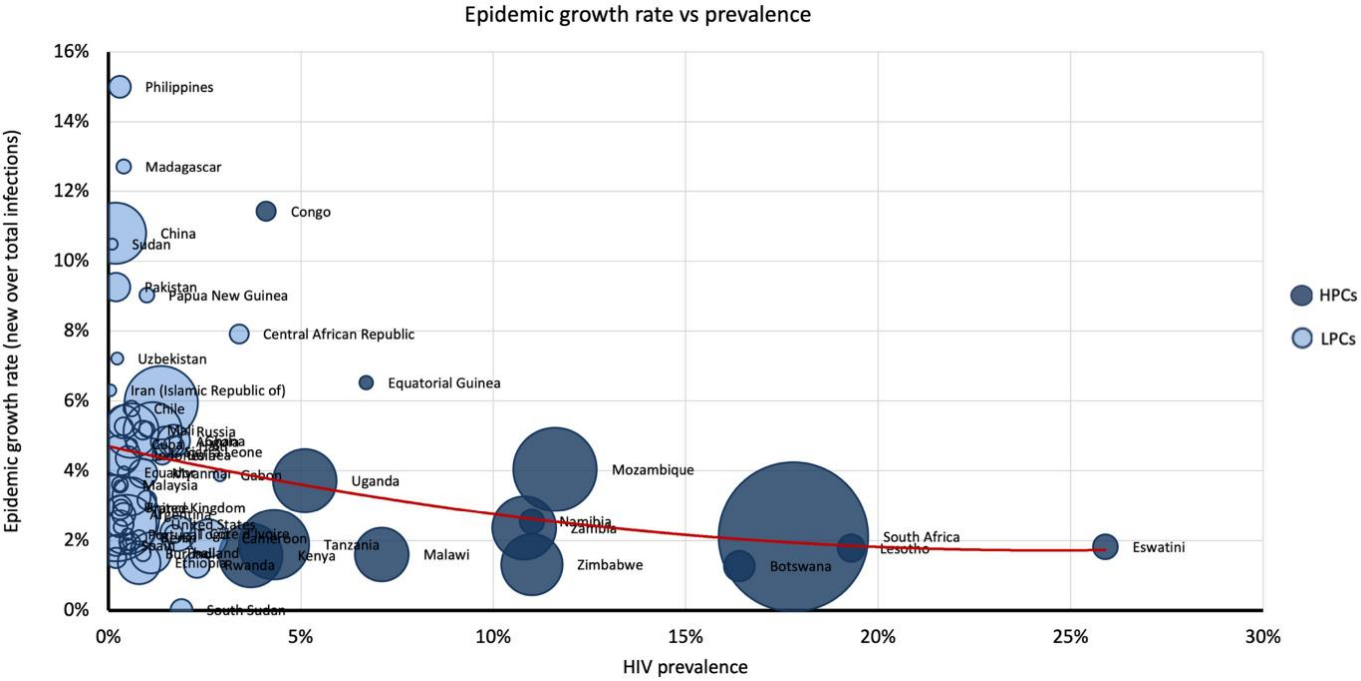
Epidemic growth rate (y-axis) versus prevalence (x-axis), weighted by epidemic size.

Figure 3 details the numbers behind this map in a bubble plot to give weighting by epidemic size. LPCs (light blue) with higher growth rates tended to have smaller prevalence, whereas HPCs (dark blue) generally conform to the x-axis indicating consistently low epidemic growth rate. The opposite is true for LPCs (light blue), which conform to the y-axis indicating consistently low prevalence but a wider spread of growth rates. Linear trend (red line) shows that LPCs tended to have higher epidemic growth rates. These nations tended to have a small epidemic size, apart from China, and prevalence of less than 1%.

### ART Coverage and PrEP

**Figure 4.**
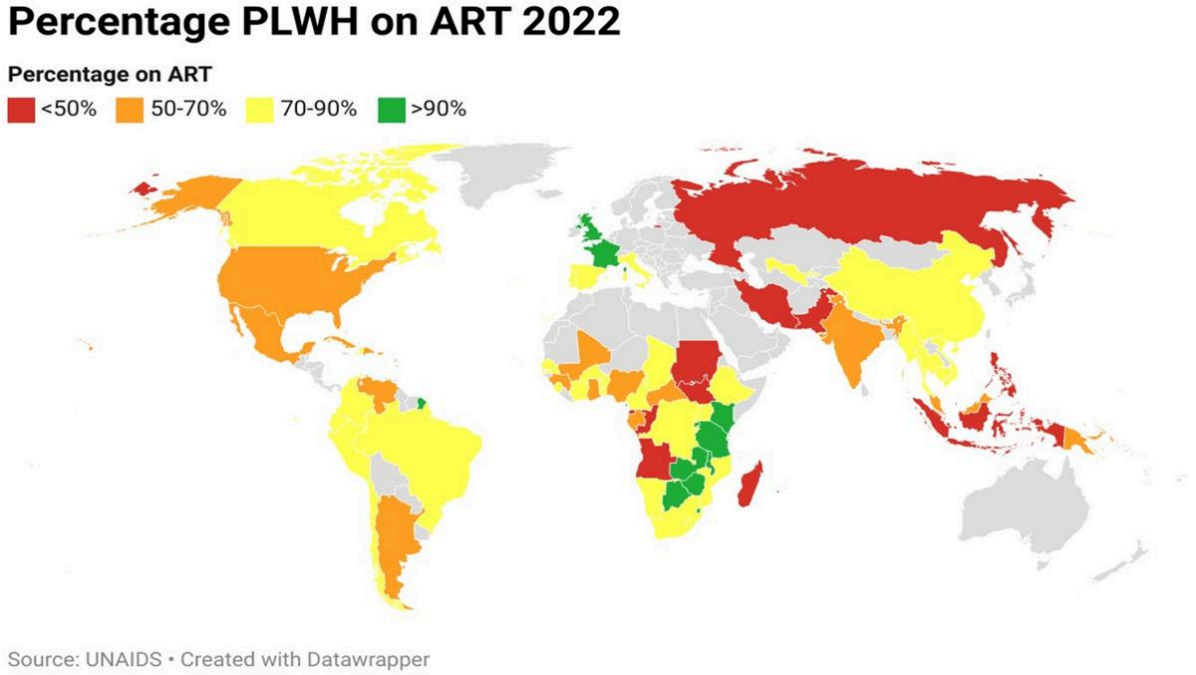
Antiretroviral therapy (ART) coverage by country.

Globally, the rate of ART coverage for PWH was 76%. In 2022, there was lower ART coverage on average in LPCs (67%) when compared to HPCs (83%). Figure 4, which differentiates nations at >90% ART coverage (10 countries), 70%–90% ART coverage (32 countries), 50%–70% ART coverage (15 countries), and <50% ART coverage (11 countries) shows that this split again defined HPCs in South-Eastern Africa as near global exceptions (green) above 90% coverage. Ten of 14 (71%) of the HPCs highlighted here were in the lower 2 quartiles for epidemic growth rate (green and yellow in figure 2). Conversely, the only LPCs achieving >90% ART coverage were France, the United Kingdom, and Rwanda. Those with poor ART coverage were predominantly Central African and Central and East Asian countries. Notably, the United States, Russia, and India also had low rates of coverage.

**Figure 5.**
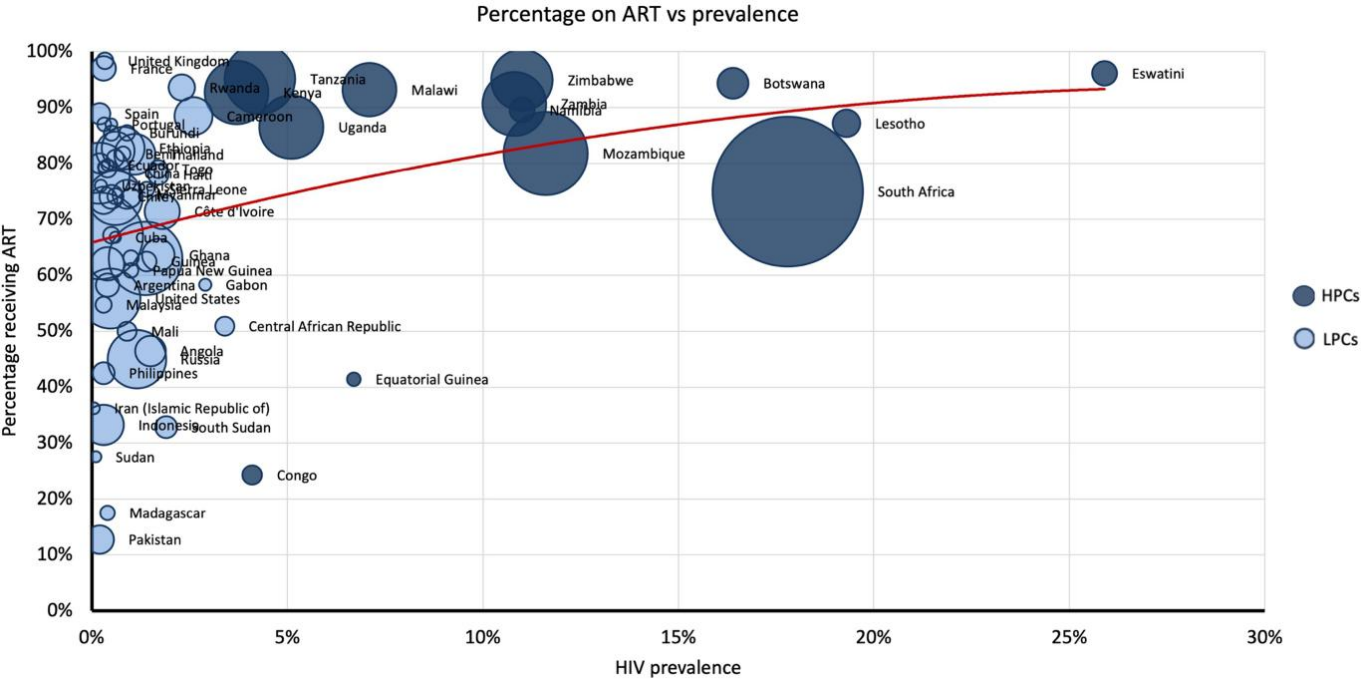
Percentage of people With HIV (PWH) receiving antiretroviral therapy (ART) (y-axis) versus prevalence (x-axis).

Figure 5 shows ART coverage against prevalence. HPCs (dark blue) conform to the upper x-axis, showing consistently high levels of ART coverage. The inverse is true for LPCs (light blue), which conform to the y-axis, indicating varying degrees of ART coverage.

PrEP was reportedly used by 1.3 million in HPCs versus 1.2 million in LPCs, though data coverage was poor. There were 770 000 new HIV infections in LPCs in 2022 and 468 000 in HPCs. Therefore, 46 million people in LPCs would have to be treated with oral PrEP to reduce incidence to 0, and a further 28 million would need to be treated in HPCs, totalling 74 million people worldwide based on number needed to treat of 60 to prevent 1 infection.

### MTCT

**Figure 6.**
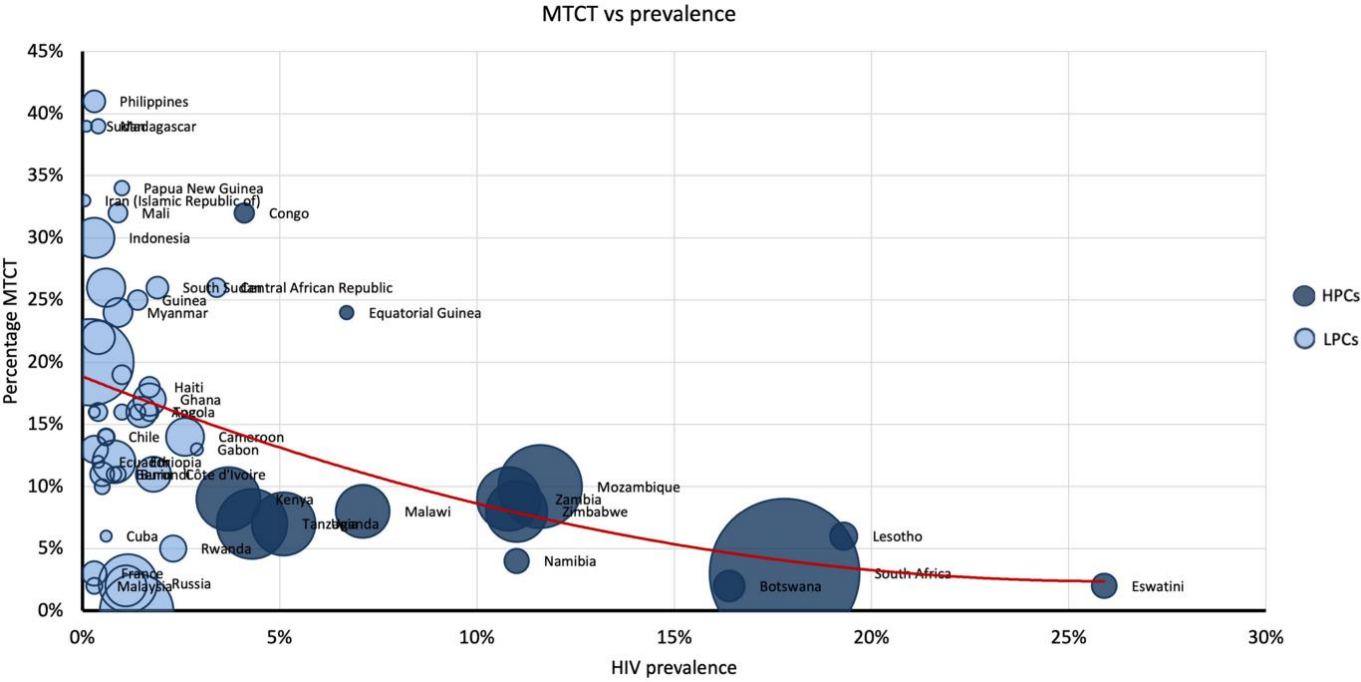
Mother-to-child-transmission (MTCT) (y-axis) versus prevalence (x-axis), weighted by epidemic size.

In concurrence with higher epidemic growth rates, LPCs had higher rates of MTCT when compared to HPCs (16% vs 9%). Figure 6 shows MTCT against prevalence. The HPCs (dark blue) conform to the x-axis showing increasing prevalence levels but consistently low MCTC rates. The LPCs (light blue) conform to the y-axis, showing consistently low prevalence but increasing rates of MTCT. The linear trend (red line) shows that countries with lower prevalence tended to have higher MTCTs.

### Case Study: Botswana

Botswana is one of the global leaders in ART delivery, with 94% coverage. As a result, the HIV epidemic growth in Botswana was well below the global average (1.3% vs 3.3%), as were AIDS-related deaths (1.1% vs 1.6%), MTCT (2% vs 11%), and AIDS-related deaths in children (0–14 years) (2.9% vs 5.6%) (Table 2). Extrapolating Botswana's 1.3% epidemic growth rate to the LPCs in this study, 550 000 new HIV infections could be prevented every year. With Botswana's 94% ART coverage globally, there would be 800 000 new infections prevented, 200 000 AIDS-related deaths prevented, 40 000 AIDS-related deaths in children aged 0–14 years, and an MTCT of 2% (Table 2).

**Table 2.** Global Epidemic Key Statistics vs Botswana, and Extrapolated Cases

	Global Number 2022	Global Rate 2022	Botswana Rate 2022	Total Difference in Global Number Using Botswana Rate
ART coverage	29 800 000	76%	94%	+6 994 664
New infections	1 300 000	3.3%	1.3%	−806 764
AIDS-related deaths	630 000	1.6%	1.1%	−194 117
AIDS-related deaths in children (0–14 y)	84 000	5.6%	2.9%	−41 142
MTCT	N/A	11%	2%	N/A

Abbreviations: ART, antiretroviral therapy; MTCT, mother-to-child-transmission; N/A, not available.

### Pharmaceutical Revenue

**Figure 7.**
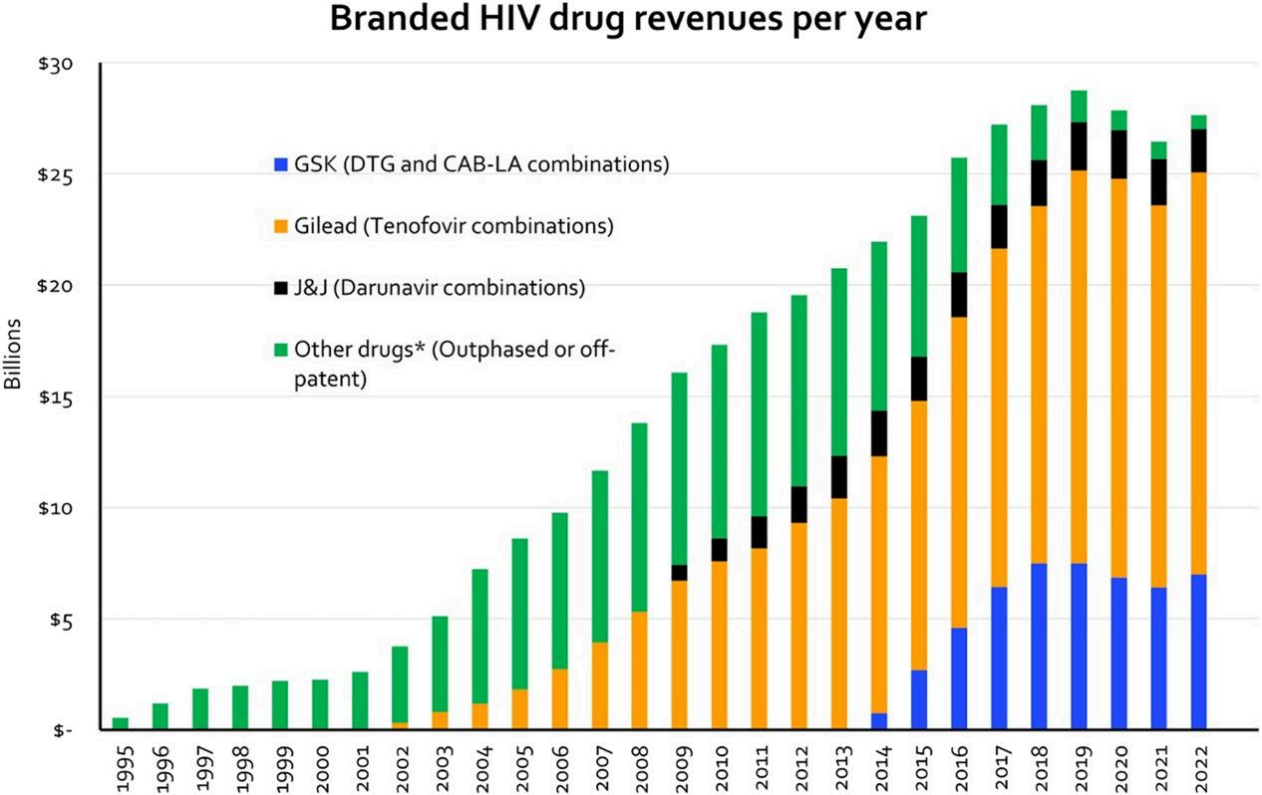
Combined annual revenue of HIV drug sales.

Figure 7 shows the annual combined revenue from HIV drugs for 3 of the main HIV drug providers. In 2022, these 3 companies generated $27 billion in annual revenue from drugs used to treat HIV. The Clinton Health Access Initiative reported a benchmark price of $42 PPPY for TLD [8]. If there were global access to ART at this price, it would cost $1.6 billion per year to treat 39 million PWH. This cost is more than 16 times smaller than the HIV-related revenue generated by 3 pharmaceutical companies in 2022.

## DISCUSSION

Worldwide, 770 703 (62%) new HIV infections are now reported in 54 countries outside of South-Eastern Africa, despite their lower total epidemic size (17 459 263) than the 14 African nations (19 462 000), meaning a higher epidemic growth rate of 4.4% versus 2.6% (Table 1). Infection rate in these LPCs may be explained by lower ART coverage than HPCs in South-Eastern Africa (67% vs 83%) and lower uptake of PrEP (1.2 million vs 1.3 million), leading to significantly more HIV-related deaths (383 000 vs 225 000) and higher rates of MTCT (16% vs 9%) (Table 1). Our results highlight an increasing need for effective preventative and treatment programs in LPCs.

There were several limitations to this study. The AIDSinfo database, from most of the data were extracted, uses the SPECTRUM model. Given the limited availability of surveillance data, SPECTRUM applies modelling approaches; over- and underestimations cannot be excluded [9]. Furthermore, where AIDSinfo data were not available, we analyzed heterogeneous national reports or peer-reviewed studies, meaning potential for differing methodology and inconsistent errors. As an illustrative paper, deeper statistical analyses were not required, but a regression analysis based on GDP or health system expenditure would be beneficial to explore why these differences have arisen and where funding may be being misdirected. Last, our results must be interpreted with caution; we are not recommending deprioritizing HPCs, but upscaling responses in neglected, middle-income LPCs.

Key strategies that have reduced infection rates and mortality in South-Eastern African HPCs were developed through community, academic, and activist means. Massive community care adjustments and targeted support through aid initiatives like PEPFAR made an exceptional difference. However, these efforts only became truly transformative when aligned with cheap and readily available ART. For this to happen, patient and civil society groups put prolonged pressure on the pharmaceutical industry. Since then, safety and efficacy of pharmaceutical prevention and treatment for HIV has steadily improved. Novel drugs are no longer needed. But research and design processes for new drugs are still used to justify rising pharmaceutical revenues: $27 billion across the main 3 for HIV technologies in 2023. Regardless, most genuinely innovative research is public [10]. Despite this, the results are exclusively licensed and commercialized, and access initiatives are limited to discretionary voluntary licensing agreements.

Previous analysis has shown that these access agreements are not justified by GDP or health system funding [2, 5]. Our current analysis shows that they are not justified by HIV infection trends, mortality distribution, or strength of treatment and prevention programs either. These agreements need to be more empirically validated and adaptive, without sharp cutoffs delineated by simple metrics such as prevalence. Many MICs in South America, West Africa, and Asia miss out on access initiatives despite high infection rates because they are below prevalence cutoffs and above GDP cutoffs set in these agreements.

Technology patenting and the resultant legislative challenges for access to medicines misdirect energy and funding from the real causes of prolonged and unequal HIV epidemics. Health equity means uninhibited health care according to need. Risk groups are often also discriminated against by existing services. The huge caseload and mortality in MICs results from avoidable and reducible health inequity. Generically available medicines are essential, and the pathway for their use must also be safe, efficient, and destigmatized. To push toward 95-95-95, LPCs can apply lessons learnt from successful campaigning and clinical activity in South-East African nations. Though vertical HIV-targeted programs can detract from wider shifts in health system strengthening [6], some mechanisms of successful HIV programs have had widespread positive influence on health outcomes that could translate to other nations, even those with stable health care systems, for example, health system decentralization to communities [7], task shifting [11], and uptake of mobile technology [12]. Transferable methods will be presented chronologically: prevention, testing, treatment, retention-in-care.

Prevention must be cheap and readily available. Total use of PrEP is far below the 74 million people required to optimize preventive efficacy. More PrEP has been delivered in 14 South-East African countries than 54 LPCs around the rest of the world. Huge incidence reduction in the Netherlands (95%) and Australia (88%) show what is possible with rapid and wide introduction of PrEP [13, 14]. Crucially, these programs provide PrEP free at the point of access. Clinical trials and incidence surveys have shown that Tenofovir Disoproxil Fumarate (TDF)/Emtricitabine (FTC) needs to be given to at least 60 people at high risk of HIV transmission to prevent 1 new infection, so free or extremely cheap access is vital.

Oral PrEP (TDF/FTC) is off-patent and available across the globe. However, roll out of PrEP has been poor, despite the Global Fund pooled procurement price of approximately 45 USD per year [8]. One year's access to generic PrEP at the appropriate scale to meet global prevention targets would cost approximately $37 million, less than one-hundredth the annual revenues of the main 3 pharmaceutical companies. Meanwhile, cabotegravir (CAB-LA) has been licensed as a long-acting injectable form of PrEP but is inaccessible for many low- and middle-income countries [8]. CAB-LA provides a different option, potentially more acceptable and less stigmatizing with a 2-monthly, pill-free regimen. Efficacy in clinical trial settings is superior to TDF/FTC [15, 16]. However, ViiV markets CAB-LA at a nonprofit price of $25 per shot, or $150 per year, versus $45 per year for generic oral PrEP [8]. Though a voluntary license for access in LMICs was reached, 38 countries were excluded from the license despite constituting 122 000 infections and lower GDP than included nations, meaning prices up to $22 200 per year [2]. Furthermore, the restrictive ViiV Medicines Patent Pool (MPP) licensing agreement means that supply is limited to a few points of production, which are saturated until 2028, meaning no access for LMICs.

Prevention is not always possible. Test-and-treat within 24 hours is the next best option, recommended by the World Health Organization (WHO). Community-based clinics and task-shifting have made this possible in South-East Africa. However, these changes are meaningless without destigmatization of HIV infection, and improved rights for gay communities and sex workers [17]. Point-of-care testing still only constitutes 30% of HIV testing [8, 18]. Low-cost, community-based testing kits are more accessible and less stigmatizing [8, 18]. These should be offered at negligible cost to at-risk communities, alongside education, especially through active recall of people known to services, for example accompanying needle-exchange programs for intravenous drug users [19]. Prices continue to fall, with many companies now offering combined home testing, though not yet approved by the WHO. MICs should focus on local production capacity or collaborative pooled procurement mechanisms like the WHO MPP to acquire high quantities of tests at low cost [20]. However, MPP usage has been limited; barriers to its use must be assessed and broken down, as seen with dolutegravir.

Countries not included in licensing deals for dolutegravir had to pay a median 8718 USD PPPY in 2018 because it is still on patent until July 2029 [21, 22]. Colombia, for example, has been forced to attempt a compulsory licence for dolutegravir [4]. Global access to generic TLD would cost $1.6 billion, 16 times less than the $27 billion revenues of the main 3 pharmaceutical companies [23–25]. Corporate responsibility to the public should be enforceable, with the expansion of march-in agreements equivalent to Bayh-Dole, allowing intervention at early stages of production and distribution.

Possibly, as debate ensues about the future of funds like PEPFAR, which supports vertical HIV programs in South-East Africa with 76% of its funding [26], ways in which international aid organizations could transform to enact deeper change rather than reciprocating outdated power dynamics should be considered. For example, PEPFAR could be composted to nurture ground-level organizations or more impactful and representative civil society groups. Restrictive licensing regimes and pharmaceutical lobbying within congress and around Trade Agreements must be challenged. At the very least, support must be negotiated for MICs no longer covered by the Global Fund or voluntary license agreements.

## CONCLUSION

LPCs now constitute more than half of the global burden of new HIV infections, mortality, and MTCT. To bring global new infections below the UNAIDS target of 370 000 per year by 2025, ART coverage needs to be optimized worldwide, and PrEP coverage expanded to 74 million people versus 2.5 million currently treated. This is possible with cheap generic TLD and TDF/FTC, for a fraction of the current global expenditure on pharmaceuticals for HIV. To meet UNAIDS 95-95-95 goals, stigma and inequality must be targeted, if the necessary funding space is to be opened up for MICs to suitably strengthen health systems and provide equitable HIV prevention and care, multilateral regulation must be reshaped so that the price of health technologies is not a barrier to progress in any nation.

## Notes

***Financial support***. This work was funded by the International Treatment Preparedness Coalition (ITPC)—Make Medicines Affordable Campaign.

***Patient consent statement*.** This paper does not include factors necessitating patient consent.

## Appendix

**Appendix 1.** Data collected for 14 high-prevalence countries (>3.5%) and 54 low-prevalence countries (<3.5%) with >40 000 people living with HIV

Country	Epidemic Size	Prevalence (%)	Percentage on ART	Incidence 2022	Epidemic Growth Rate	MTCT	AIDS-related Deaths	Income Band	Data Source
High-prevalence Countries (>3.5%)									
Eswatini	220 000	25.90%	96.1%	4000	1.8%	2%	2700	Lower-middle	UNAIDS
Lesotho	270 000	19.30%	87.2%	4800	1.8%	6%	4000	Lower-middle	UNAIDS
South Africa	7 600 000	17.80%	75.0%	160 000	2.1%	3%	45 000	Upper-middle	UNAIDS
Botswana	340 000	16.40%	94.3%	4300	1.3%	2%	3800	Upper-middle	UNAIDS
Mozambique	2 400 000	11.60%	81.8%	97 000	4.0%	10%	48 000	Low	UNAIDS
Namibia	220 000	11.00%	89.6%	5600	2.5%	4%	3100	Upper-middle	UNAIDS
Zimbabwe	1 300 000	11.00%	94.9%	17 000	1.3%	8%	20 000	Lower-middle	UNAIDS
Zambia	1 400 000	10.80%	90.7%	33 000	2.4%	9%	19 000	Lower-middle	UNAIDS
Malawi	1 000 000	7.10%	93.2%	16 000	1.6%	8%	12 000	Low	UNAIDS
Equatorial Guinea	72 000	6.70%	41.4%	4700	6.5%	24%	2800	Upper-middle	UNAIDS
Uganda	1 400 000	5.10%	86.5%	52 000	3.7%	7%	17 000	Low	UNAIDS
Tanzania	1 700 000	4.30%	95.1%	32 000	1.9%	7%	22 000	Lower-middle	UNAIDS
Congo	140 000	4.10%	24.3%	16 000	11.4%	32%	7700	Lower-middle	UNAIDS
Kenya	1 400 000	3.70%	92.7%	22 000	1.6%	9%	18000	Lower-middle	UNAIDS
Low-prevalence Countries (<3.5%)									
Central African Republic	120 000	3.40%	50.9%	9500	7.9%	26%	4500	Low	UNAIDS
Gabon	49 000	2.90%	58.3%	1900	3.9%	13%	1800	Upper-middle	UNAIDS
Cameroon	480 000	2.60%	88.5%	9900	2.1%	14%	10 000	Lower-middle	UNAIDS
Rwanda	230 000	2.30%	93.6%	3000	1.3%	5%	2700	Low	UNAIDS
South Sudan	160 000	1.90%	32.9%	11	0.0%	26%	7600	Low	UNAIDS
Côte d'Ivoire	410 000	1.80%	71.4%	9000	2.2%	11%	10 000	Lower-middle	UNAIDS
Ghana	350 000	1.70%	63.6%	17 000	4.9%	17%	9400	Lower-middle	UNAIDS
Haiti	140 000	1.70%	78.0%	6600	4.7%	18%	1600	Lower-middle	UNAIDS
Togo	110 000	1.70%	79.0%	2400	2.2%	16%	2400	Low	UNAIDS
Angola	310 000	1.50%	46.5%	15 000	4.8%	16%	13 000	Lower-middle	UNAIDS
Guinea	130 000	1.40%	62.4%	5800	4.5%	25%	3500	Lower-middle	UNAIDS
Sierra Leone	77 000	1.40%	75.3%	3500	4.5%	16%	2300	Low	UNAIDS
Nigeria	1 800 000 (2019)	1.37%	63.0%	107 112 (2019)	6.0%	–	45894 (2019)	Lower-middle	UNAIDS
Russia	1 137 000	1.15%	45.0%	58 340	5.1%	2.2%	34 000	upper-middle	HIV Russia. [Accessed from: https://tochno.st/problems/hiv]
Thailand	560 000	1.10%	81.6%	9200	1.6%	2%	11 000	upper-middle	UNAIDS
Chad	120 000	1.00%	73.9%	3800	3.2%	19%	2800	Low	UNAIDS
Dominican Republic	79 000	1.00%	63.1%	4100	5.2%	16%	1500	Upper-middle	UNAIDS
Papua New Guinea	72 000	1.00%	60.9%	6500	9.0%	34%	1100	Lower-middle	UNAIDS
Burundi	80 000	0.90%	85.5%	1300	1.6%	11%	1300	Low	CEIC Data [Accessed from: https://www.ceicdata.com/en/colombia/social-health-statistics/co-antiretroviral-therapy-coverage--of-people-living-with-hiv]
Mali	120 000	0.90%	50.0%	6200	5.2%	32%	4900	Low	UNAIDS
Myanmar	280 000	0.90%	74.5%	11 000	3.9%	24%	6400	Lower-middle	UNAIDS
Benin	72 000	0.80%	81.7%	1500	2.1%	11%	1900	Lower-middle	UNAIDS
Ethiopia	610 000	0.80%	82.7%	8300	1.4%	12%	11000	Low	UNAIDS
Brazil	990 000	0.60%	73.8%	51 000	5.2%	–	13000	Upper-middle	UNAIDS
Burkina Faso	97 000	0.60%	80.9%	1900	2.0%	14%	2600	Low	UNAIDS
Chile	83 000	0.60%	74.1%	4800	5.8%	14%	566 (2019)	High	Statista
Cuba	42 000	0.60%	66.8%	2000	4.8%	6%	500	Upper-middle	UNAIDS
Democratic Republic of the Congo	490 000	0.60%	82.3%	16 000	3.3%	26%	12 000	Low	UNAIDS
Cambodia	76 000	0.50%	85.4%	1400	1.8%	10%	1100	Lower-middle	UNAIDS
Colombia	190 000	0.50%	74.0%	8300	4.4%	11%	1500	Upper-middle	UNAIDS
Portugal	46 000	0.50%	87.0%	1000	2.2%	–	500	High	UNAIDS
Venezuela	92 000	0.50%	67.2%	1800	2.0%	–	1500	Lower-middle	GNI [Accessed from: https://publications.iadb.org/en/venezuela-still-upper-middle-income-country-estimating-gni-capita-2015–2021]
United States	1 200 000	0.47%	55.8%	29 500	2.5%	–	18 489 (2020)	High	CDC [Accessed from: https://www.hiv.gov/hiv-basics/overview/data-and-trends/statistics]
Argentina	182 041	0.40%	58.2%	5000	2.7%	–	1300	Upper-middle	Index Mundi [https://www.indexmundi.com/facts/argentina/hiv-incidence]
Ecuador	48 000	0.40%	79.7%	1900	4.0%	12%	500	Upper-middle	UNAIDS
Madagascar	70 000	0.40%	17.5%	8900	12.7%	39%	3200	Low	UNAIDS
Mexico	370 000	0.40%	62.0%	20 000	5.4%	22%	4600	Upper-middle	UNAIDS
Peru	110 000	0.40%	79.1%	5800	5.3%	16%	1000	Upper-middle	UNAIDS
United Kingdom	91 432	0.33%	98.4%	2692	2.9%	–	723	High	UK GOV [Accessed from: https://www.gov.uk/government/statistics/hiv-annual-data-tables]
Canada	62 790	0.31%	87.0%	1500	2.4%	–	1538	High	Government of Canada [Accessed from: https://www.canada.ca/en/public-health/services/publications/diseases-conditions/estimates-hiv-incidence-prevalence-canada-meeting-90–90–90-targets-2020.html]
France	200 000	0.30%	97.0%	5900	3.0%	3%	1000	High	UNAIDS
Indonesia	540 000	0.30%	33.3%	24 000	4.4%	30%	26 000	Upper-middle	UNAIDS
Malaysia	86 000	0.30%	54.7%	3100	3.6%	2%	2500	Upper-middle	Code Blue [Accessed from: https://codeblue.galencentre.org/2022/12/06/hiv-stigma-in-malaysia-still-very-much-alive-in-2022/]
Philippines	160 000	0.30%	42.5%	24 000	15.0%	41%	1500	Lower-middle	UNAIDS
Senegal	42 000	0.30%	79.4%	1500	3.6%	16%	1000	Lower-middle	UNAIDS
Viet Nam	250 000	0.30%	73.4%	6200	2.5%	13%	4100	Lower-middle	UNAIDS
Uzbekistan	48 000	0.23%	76.0%	3463	7.2%		680	Lower-middle	World bank [Accessed from: https://data.worldbank.org/country/uzbekistan]
India	2 500 000	0.20%	67.0%	66 000	2.6%	20%	40 000	Lower-middle	UNAIDS
Italy	140 000	0.20%	80.0%	2100	1.5%	–	1000	High	UNAIDS
Pakistan	270 000	0.20%	12.7%	25 000	9.3%	–	12 000	Lower-middle	WHO [Accessed from https://www.emro.who.int/asd/country-activities/pakistan.html]
Spain	150 000	0.20%	88.9%	2785	1.9%	–	1000	High	UNAIDS
China	1 250 000	0.19%	78.3%	135 000	10.8%	–	34 800	Upper-middle	Bavinton et al, 2021 [Accessed from: https://www.thelancet.com/journals/lanpub/article/PIIS2468–2667(21)00112–2/fulltext]
Sudan	41 000	0.10%	27.6%	4300	10.5%	39%	100	Low	UNAIDS
Iran (Islamic Republic of)	46 000	0.05%	36.2%	2900	6.3%	33%	2300	Lower-middle	UNAIDS

Non-UNAIDS sources are listed in column K. Non-UNAIDS data are highlighted in red.

Abbreviations: ART, antiretroviral therapy; MTCT, mother-to-child transmission; UNAIDS, United Nations Program and HIV/AIDS; WHO, World Health Organization.
